# Minimally invasive (sinus tarsi) approach for calcaneal fractures

**DOI:** 10.1186/s13018-016-0497-4

**Published:** 2016-12-23

**Authors:** Zhe Wang, Xiu Hui Wang, Sheng Long Li, Xin Tang, Bei Gang Fu, Ming Hui Wang, Sheng Li Xia

**Affiliations:** 1 Department of Orthopedic Trauma, The First Affiliated Hospital of Dalian Medical University, 222, Rd Zhongshan, Xigang District, Dalian, Liaoning Province 201318 China; 2 Department of Orthopedics, Shanghai Zhoupu Hospital, Pudong New Area 1500, Rd Zhouyuan, Zhoupu, Shanghai, 201318 China; 3 Department of Orthopedics, Dalian Central Hospital, Dalian Medical University, Dalian, China

**Keywords:** Calcaneal fractures, Sinus tarsi, Combined plate fixation

## Abstract

**Background:**

According to the anatomic characteristics of the calcaneus and the sinus tarsi approach, we designed a combined plate. The goal of this study was to retrospectively assess the functional outcomes and complications of treatment with our self-designed plate.

**Methods:**

From March 2014 to October 2015, 18 patients with closed calcaneal fractures (14 Sanders type II and 4 type III) were treated with our combined locking plate through a minimally invasive sinus tarsi approach. All patients underwent both clinical and radiological evaluations.

**Results:**

The follow-up duration for all patients ranged from 6 to 13.5 months. The radiographs demonstrated significant corrections of the calcaneal width, length, height, Böhler angle, and Gissane angle from preoperatively to 3 months postoperatively and the last follow-up. However, there were no significant differences in the variables between 3 months postoperatively and the last follow-up. The mean Maryland foot score was 88.1 ± 8.8, in which excellent outcomes were achieved in 11 patients, good in 4, and fair in 3 (excellent and good rate, 83.3% (15 of 18)). No statistical significances in the mean Maryland foot score (88.1 ± 8.8 vs 87.8 ± 10.1, *p* = 0.9), and the excellent and good rate (85.7 vs 75.0%, *p* = 1.0) was found between type II and type III fractures. No complications were observed in all fractured feet.

**Conclusion:**

Treatment with our self-designed combined plate through a sinus tarsi approach may be safe and effective for type II and type III calcaneal fractures.

**Electronic supplementary material:**

The online version of this article (doi:10.1186/s13018-016-0497-4) contains supplementary material, which is available to authorized users.

## Background

Fractures of the calcaneus are commonly encountered clinical injuries resulting from high-energy trauma. They account for 1 to 2% of all fractures and 60% of all tarsal fractures. According to the results of the computed tomography (CT) scanning, the calcaneal fractures can be classified into four categories, among which the Sanders types II and III fractures are the most common types [1]. Thus, the development of effective and safe treatment strategies for these two fracture types has always been a hot issue among orthopaedic surgeons [2].

Currently, open reduction and internal fixation through the lateral L-shape extensile incision has been considered as the gold standard surgical therapy for calcaneal fractures. This approach provides a large view to expose the fractures, allowing accurate reduction of the deformed posterior facet and convenient placement of the plate to achieve a stable fixation. However, the high incidence (approximately 30%) of complications associated with this approach, including wound dehiscence and deep infection, remains a non-negligible problem [3, 4].

To lower the wound complications, a minimal incision approach at the sinus tarsi has been proposed. Several randomized controlled trials have demonstrated a similar reduction; however, a significantly decreased risk of wound complications can be obtained with this procedure in comparison with the lateral extended approach [5, 6]. Nevertheless, the poor visualization of the lateral wall of the calcaneus through this small incision makes it difficult to insert the conventional plate for obtaining a stable fixation. Thus, the development of a plate that is adaptable to the anatomic characteristics of the calcaneus and sinus tarsi approach is important [6, 7].

In the present study, we self-designed a new, combined, anatomical plate specifically for Sanders type II and type III fractures and aimed to retrospectively assess the functional outcomes and complications of its use through the sinus tarsi approach.

## Methods

### Patients

This study was approved by the Institutional Review Board of the First Affiliated Hospital of Dalian Medical University, and all patients provided written informed consent for the surgery and for this study. We retrospectively reviewed the clinical data of patients with calcaneal fractures who were treated by using a minimally invasive sinus tarsi approach in the First Affiliated Hospital of Dalian Medical University between March 2014 and October 2015. The eligible patients met the following inclusion criteria: (1) age >18 years; (2) with closed fracture of the calcaneus; (3) with Sanders types II or III fractures [1]; (4) with fractures fixed by using our newly self-designed combined locking plate; and (5) with a minimum of 6 months of clinical follow-up. Patients with history of calcaneal, ankle, or other foot fractures were excluded.

### Our self-designed combined plate

**Fig. 1:**
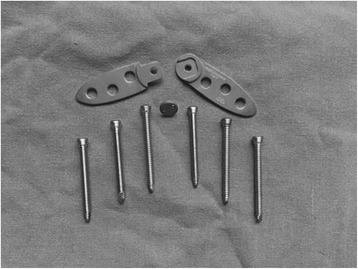
Structure of our self-designed combined plate

The plate (GJPS(I)67; 1.5 mm in thickness and 13 mm in width; made of titanium alloy owing to the better biocompatibility, flexibility, and a lower resonance of this material [8]; Puwei Medical Instrument Co., Ltd, Shanghai, China; patent no. ZL 2014 2 0269654.X) consisted of two sheets, mutually independent fixed arms (forearm, 42 mm in length; rear arm, 39 mm in length), a coupling screw, and six locking screws (Fig. 1). There are three screw holes in the forearm/rear arm to lockingly fix the anterior process of the calcaneus, calcaneal body, and bones behind the calcaneal body, respectively. The head end of the forearm/rear arm is of streamlined shape, whereas the tail end can be linked by the coupling screw through their chimeric structure.

### Analysis of the biomechanical properties of our combined plate

**Fig. 2:**
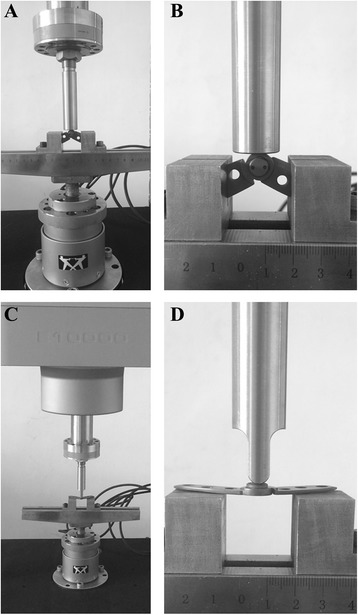
The biomechanical testing for the plate. **a**, **b** Vertical load-bearing test. **c**, **d** Resistance to bending load test

The biomechanical characteristics of our self-designed combined plate were tested by Biomechanics Laboratory of Changzhou Waston Medical Appliance Company (Changzhou, China) by using the standard electro-mechanical testing machine (Instron ElectroPuls E10000; Instron Systems, Norwood, MA, USA) equipped with a 10-kN Instron load cell. The vertical load-bearing capacity of the combined plate was tested under static (in which the loading rate was 2 mm/min and the maximum load was recorded by monitoring the force-displacement curves) and dynamic (performed in load-controlled mode with sinusoidal wave at a frequency of 5 Hz and maximum load of 500 N) conditions (Fig. 2a, b). For the resistance to bending loads, the force-displacement curve was also monitored and the load to failure was recorded at the loading velocity of 2 mm/min and with the span of 30 mm (Fig. 2c, d).

### Surgical procedure

All procedures were performed by the same surgeon (Zhe Wang) as previously described [6, 7, 9]. After admission, the affected feet of the patients were fixed by using a plaster or provisional brace and elevated to prevent the exacerbation of injuries in the soft tissues that can be induced by activity. For patients with slight damages to the soft tissue, the surgery was scheduled within 48–72 h; however, for patients with severe tissue damages (accompanied by obvious swelling and tension blisters), the surgery was arranged after 7–10 days to confirm the reduction of swelling, improvement of soft tissue condition, and a positive wrinkle test. This led to a mean interval from injury to surgery of 4.7 ± 2.4 days (range, 2–9 days).

**Fig. 3:**
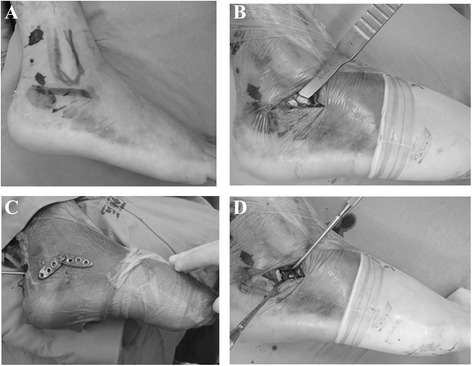
Intraoperative pictures of the sinus tarsi approach. **a** Marking for the sinus tarsi approach (*solid line*). **b** Incision over the sinus tarsi and subcutaneous tunnel created by using a small periosteal elevator. **c** Schematic diagram of plate placement. **d** Placement of our self-designed combined plate through the sinus tarsi approach

Surgery was performed with the patients placed in a lateral decubitus position under epidural or subarachnoid anaesthesia (Additional file 1). An electric pneumatic tourniquet (pressure, 45 KPa) was used on the affected limb to reduce blood loss and improve the operative field visually. One or two Steinmann pins were first drilled from the calcaneus to the head of the talus to correct the Böhler angle and restore the Gissane angle, which was confirmed under C-arm fluoroscopy. Then, a 3–4-cm incision was made over the sinus tarsi (beginning from the tip of the lateral malleolus to the proximal cuboid) to expose the lateral cortex of the anterior process of the calcaneus (carefully, to avoid injuring the lateral sural cutaneous nerve, peroneus longus, and brevis tendon) (Fig. 3a, b). Subsequently, a subcutaneous tunnel was created by using a small periosteal elevator in the above incision, and our self-designed plate was inserted to the corresponding position of the lateral wall of the calcaneus, which was temporarily held in place with two Kirschner wires (Fig. 3c, d). After a satisfactory reduction was achieved under C-arm fluoroscopy, six locking screws and a coupling screw were screwed followed by the removal of the Steinmann pin and closure of the incision in layers.

### Postoperative management and evaluation

Postoperatively, prophylactic antibiotics were given to prevent surgical site infection and plaster immobilization was performed to protect the wound. The patients were encouraged to do toe flexion and dorsiflexion exercises 24 h after the operation. Stitches were removed after 2 weeks of surgery, and weight bearing was gradually advanced to regain full range of motion and strength at 4 weeks after surgery, during which the plaster cast was removed.

The operative data were recorded, including operative time (min; measured from the cut to the suture of the incision), hospital stay (days), cumulative intraoperative radiation time (min; provided by the fluoroscopic apparatus), fracture healing time (days), and the incidence of complications. Fracture healing was defined as the bridging of the trabecular bone and the disappearance of the trabecular fracture line on the radiograph. The lateral and axial radiographs were also obtained preoperatively, postoperatively, and at the last follow-up to judge the reduction of the calcaneus, including the Böhler angle (i.e. the angle between the line drawn from the highest point of the anterior process to the highest point of the posterior facet and the line tangent to the tuberosity); Gissane angle (i.e. the angle between the line tangent to the articular surface of the medial posterior facet and the line of the anterior end of the posterior facet to the dorsal edge of the calcaneocuboid facet); calcaneal height (i.e. the perpendicular distance from the inferior cortex of the calcaneus to the top of the medial posterior facet); calcaneal width (i.e. the distance between the external cortex of the medial malleolus and the lateral cortex of the most lateral calcaneal fracture fragment); and calcaneal length (i.e. the orthogonal distance from the most posterior aspect of the calcaneus to the most distal edge of the calcaneocuboid joint) [10, 11]. The clinical functional outcomes were assessed by using the Maryland foot score (MFS), which is a 100-point scoring system with 40 points for function, 45 points for pain, 10 points for cosmesis, and 5 points for movement of the ankle, subtalar, midfoot, and metatarsophalangeal joints. The scores were defined as follows: 90–100 excellent, 75–89 good, 50–74 fair, and <50 poor [12]. All data collection and measurement procedures were performed by Zhe Wang and checked by Sheng Long Li. Measurement of reduction parameters was accomplished with the DWRuler software.

### Statistical analysis

All data are expressed as *n* (%) or mean ± standard deviation (SD) and analysed by using SPSS 18.0 statistical software (SPSS Inc., Chicago, IL, USA). The preoperative and postoperative radiological results were compared by using paired Fisher’s exact test (because of an expected frequency of <5), and an independent *t* test was used to compare the functional outcomes between the different Sanders classifications. A value of *p* < 0.05 was considered to indicate a significant difference.

## Results

### Patient demographic and fracture characteristics

From March 2014 to October 2015, 18 patients (14 men, 4 women) with calcaneal fractures were enrolled in this study. Their average age at the time of the calcaneal fracture was 50.4 ± 10.1 years (range, 32–66 years). The mechanism of injury was falling from a height in 14 cases, traffic accidents in 2 cases, strike injury in 1 case, and others in 1 case. All fractures were unilateral, with the left side involved in 7 cases and the right side in 11 cases. In addition, the lumbar vertebral, clavicular, and ankle fractures were complicated in 1 case. Six patients had a >5 years smoking history and one patient had a history of excessive consumption of alcohol, and they were advised to stop smoking and abstain from alcohol until wound healing; on the other hand, two patients had diabetes, and their blood glucose was controlled to prevent complications [13, 14]. According to the Sanders CT scan classification system, there were 14 feet with type II fractures (8 type IIA, 4 type IIB, and 2 type IIC) and 4 feet with type III fractures (2 type IIIAB, 1 type IIIAC, and 1 type IIIBC) (Table 1, Additional file 2: Table S1) [1].

**Table 1:** Demographic data of 18 patients with calcaneal fractures

Characteristic	Frequency count (%) or mean ± SD
Sex	
Female	4 (22.2)
Male	14 (77.8)
Age (year)	50.4 ± 10.1
Side	
Left	7 (38.9)
Right	11 (61.1)
Injury mechanism	
Falling from a height	14 (77.8)
Traffic accidents	2 (11.1)
Stroke	1 (5.6)
Others	1 (5.6)
Sanders classification	
Type IIA	8 (44.4)
Type IIB	4 (22.2)
Type IIC	2 (11.1)
Type IIIAB	2 (11.1)
Type IIIAC	1 (5.6)
Type IIIBC	1 (5.6)
Complicated other fractures	
Lumbar vertebral	1 (5.6)
Ankle	1 (5.6)
Clavicular	1 (5.6)
History	
Smoking	6 (33.3)
Diabetes	1 (5.6)
Excessive consumption of alcohol	2 (11.1)

### Biomechanical outcomes

**Fig. 4:**
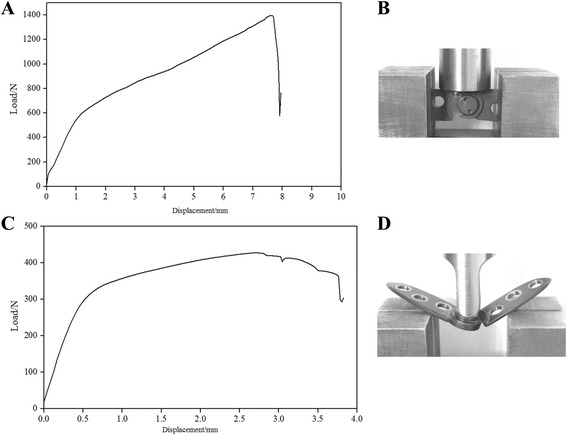
Representative curve for biomechanical testing of our combined plate. A gradual increase in stress with a sudden decrease in displacement indicated structural failure. **a** Curve for the vertical load-bearing test. **c** Curve for the resistance to bending load test. **b**, **d** Plate breakage

The analysis of biomechanical properties demonstrated that the plate could bear 1396.03 N in maximum vertical load-bearing capacity and 427.15 N in maximum resistance to bending loads (Fig. 4). Under a cyclic loading of 500 N, plate breakage was observed after 93,003 cycles.

### Surgical outcomes

**Fig. 5:**
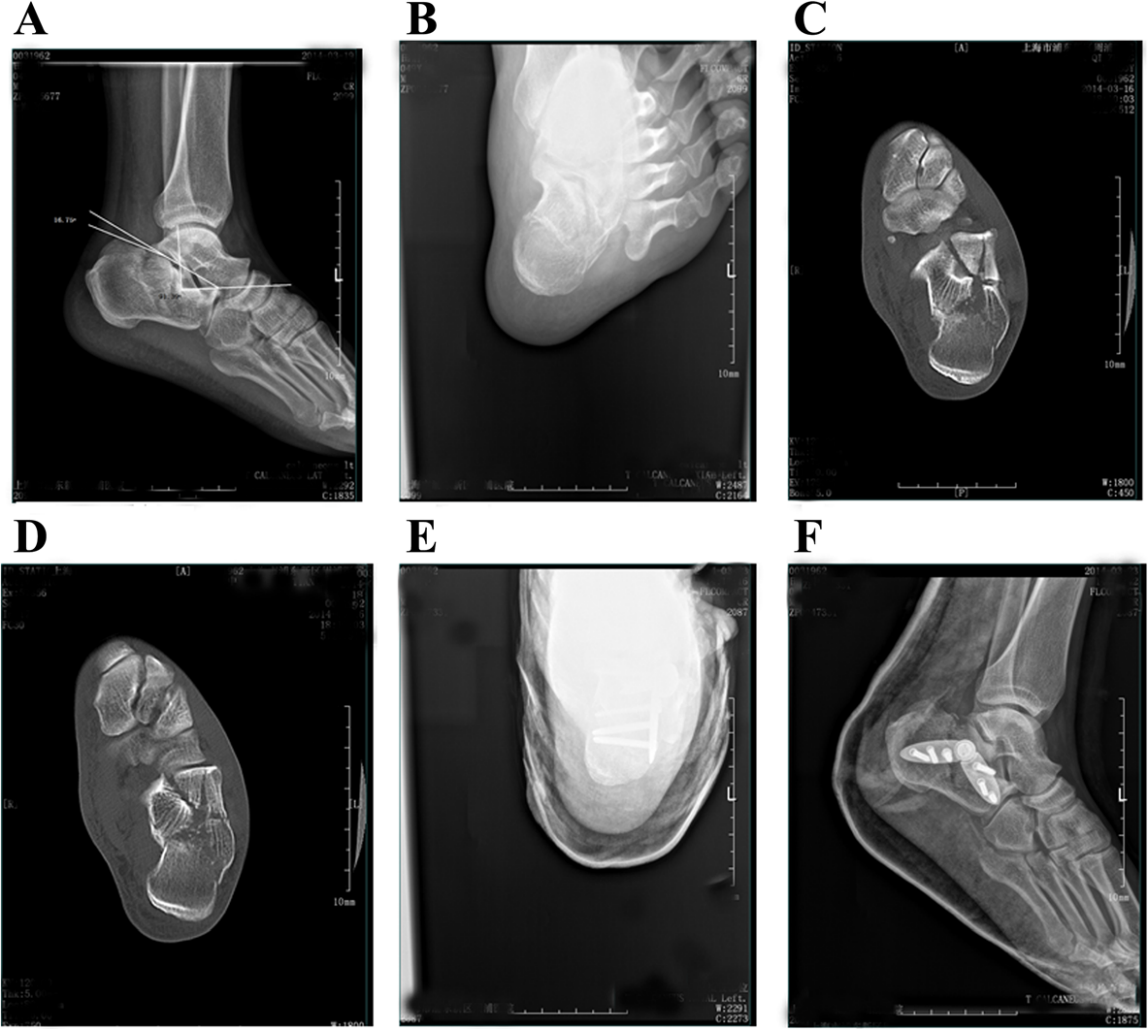
A 52-year-old male patient was admitted to our hospital because of pain and swelling on the right foot after falling from a height. Preoperative lateral (**a**) and axial (**b**) radiographs showing the right calcaneal fracture, with reduced Böhler angle (16.75°), Gissane angle (91.39°), calcaneal length (58.23 mm), and calcaneal height (38.59 mm) but increased calcaneal width (38.59 mm). Preoperative horizontal (**c**) and coronal (**d**) computed tomography images showing a Sanders IIC fracture. Postoperative lateral (**e**) and axial (**f**) radiographs showing obvious corrections of the Böhler angle (23.17°) and Gissane angle (124.4°) and improvements in calcaneal length (70.28 mm), height (35.6 mm), and width (35.54 mm)

**Fig. 6:**
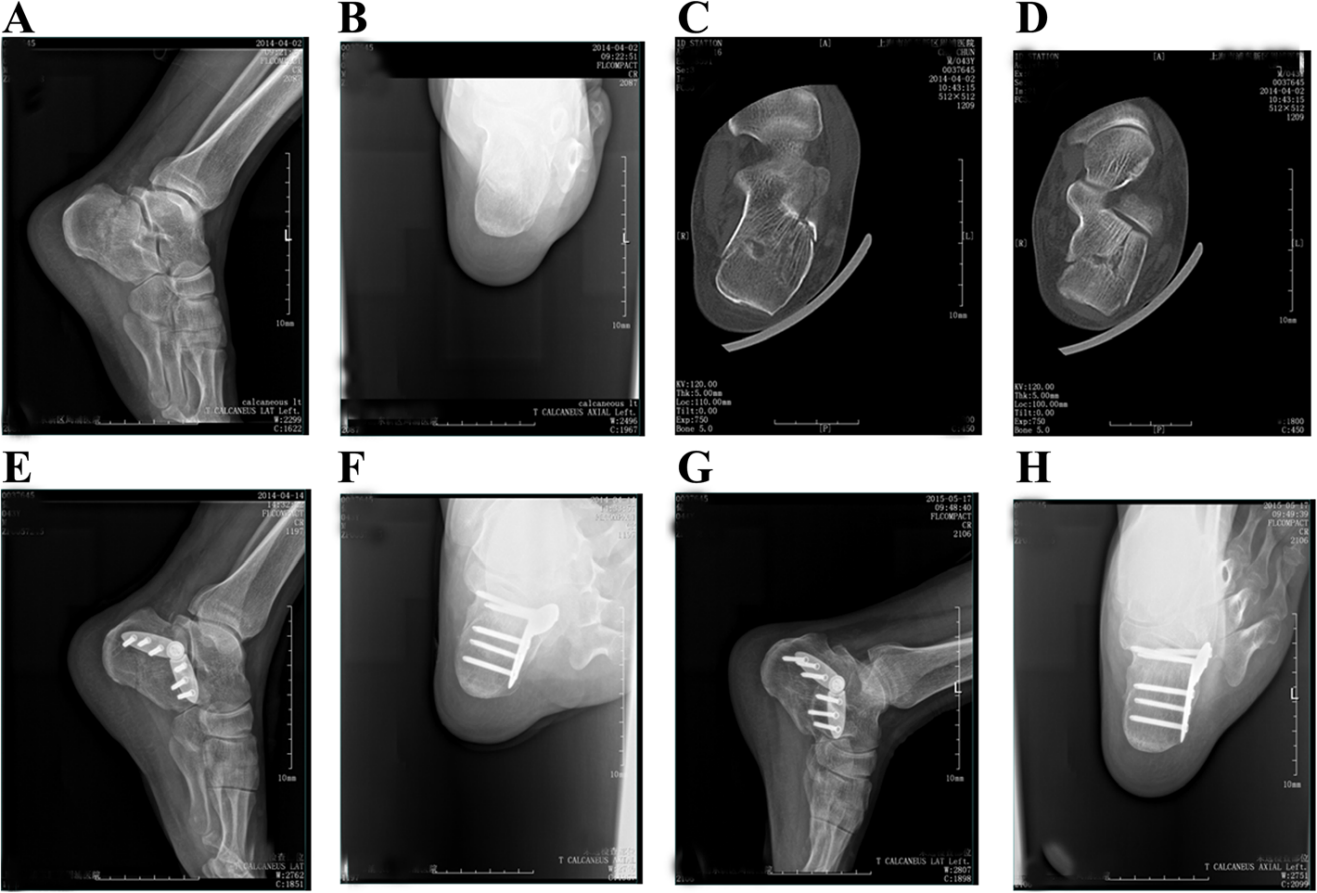
A 45-year-old male patient was admitted to our hospital because of pain and swelling on the left foot after falling from a height. Preoperative lateral (**a**) and axial (**b**) radiographs showing the left calcaneal fracture, with reduced calcaneal height (34.5 mm) and length (62.3 mm) but increased calcaneal width (39.4 mm). Preoperative horizontal (**c**) and coronal (**d**) computed tomography images showing a Sanders IIA fracture with a bulked lateral wall. Postoperative lateral (**e**) and axial (**f**) radiographs showing obvious corrections of the calcaneal height (38.6 mm), length (67.5 mm), and width (39.3 mm). Postoperative lateral (**g**) and axial (**h**) radiographs showing excellent fracture union, with a Maryland foot score of 88

**Fig. 7:**
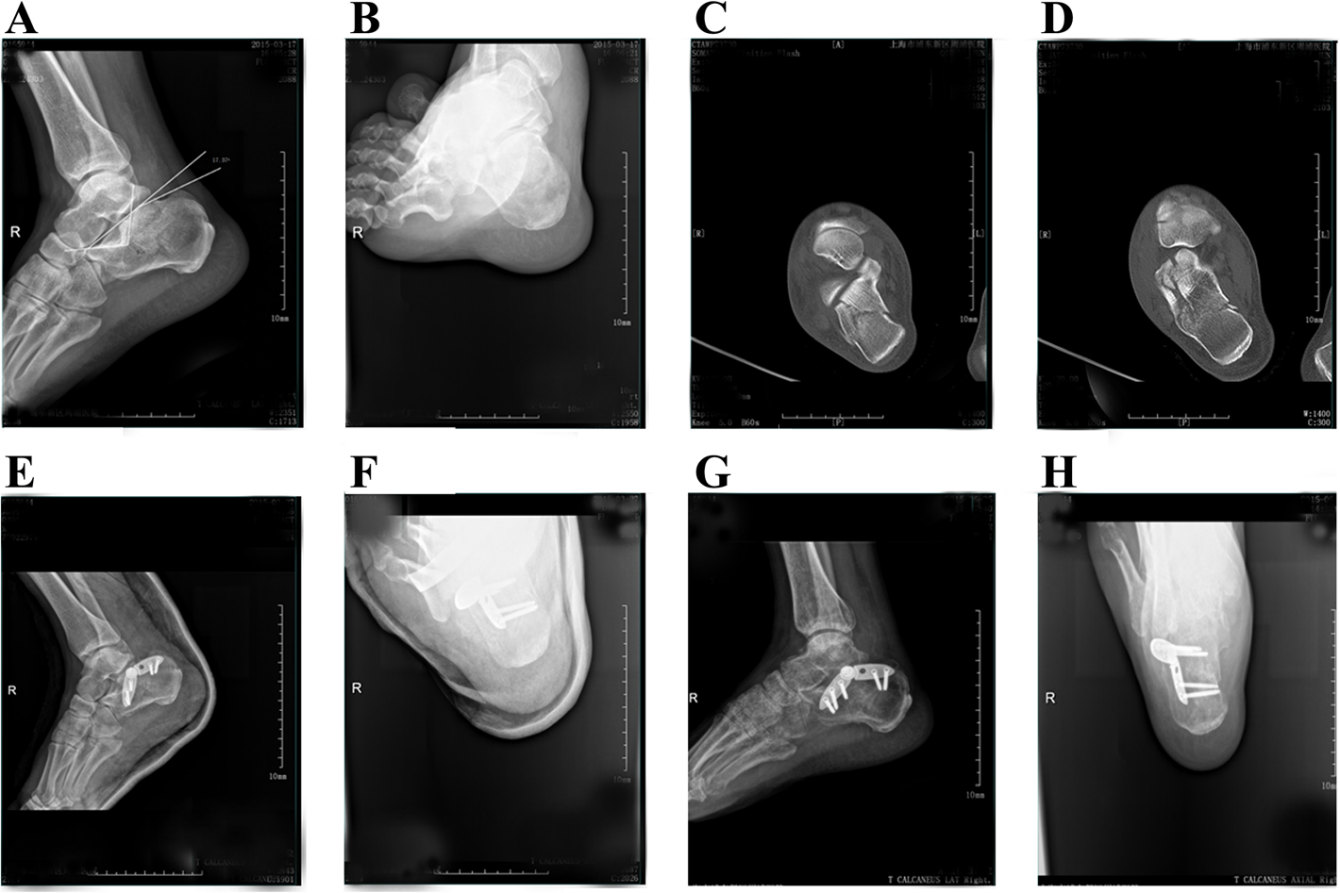
A 41-year-old male patient was admitted to our hospital because of pain and swelling on the right foot after falling from a height. Preoperative lateral (**a**) and axial (**b**) radiographs showing the right calcaneal fracture, with reduced Böhler angle (17.37°) and Gissane angle (97°). Preoperative horizontal (**c**) and coronal (**d**) computed tomography images showing a Sanders IIA fracture with a bulked lateral wall. Postoperative lateral (**e**) and axial (**f**) radiographs showing obvious corrections of the Böhler angle (23.17°) and Gissane angle (124.4°). Postoperative lateral (**g**) and axial (**h**) radiographs showing excellent fracture union, with a Maryland foot score of 87

The surgery was successful in all patients, with a mean operative time of 64.4 ± 8.0 min (range, 50–80 min), mean radiation time of 13.0 ± 3.5 s (range, 8–21 s), and mean hospital stay of 8.9 ± 2.2 days (range, 5–13 days). The radiographs showed that all fractures healed uneventfully, with a mean time to bone union of 89.3 ± 11.0 days (range, 75–113 days). The radiographs also demonstrated adequate reduction of the fracture (Table 2, Additional file 2: Table S1), with the calcaneal width, length, height, Böhler angle, and Gissane angle significantly corrected from preoperatively to 3 months postoperatively and the last follow-up (range, 6–13.5 months; mean, 9.3 ± 3.7 months). However, there were no significant differences when comparing the calcaneal width, length, height, Böhler angle, and Gissane angle between 3 months after the operation and at the last follow-up, indicating that this reduction effect can be maintained but not lost during follow-up. The overall mean MFS was 88.1 ± 8.8, in which excellent outcomes were achieved in 11 patients, good in 4, and fair in 3 (excellent and good rate, 83.3% (15 of 18)). Further comparison between type II and type III fractures showed no statistical significances in the mean MFS (88.1 ± 8.8 vs 87.8 ± 10.1, *p* = 0.9) and in the excellent and good rate (85.7 vs 75.0%, *p* = 1.0), suggesting that our self-designed plate may be equally effective for these two types of fractures (Table 3, Additional file 2: Table S1). Moreover, no screw loosening and screw or plate breakage was observed, and none of the patients developed incision infection, poor wound healing, and other complications during the follow-up, proving the favourable safety profile of the plate. Radiographs of three typical cases (case 1, Fig. 5a–f; case 2, Fig. 6a–h; case 12, Fig. 7a–h) were provided to illustrate the preoperative and postoperative changes.

**Table 2:** Radiological results before and after operation

Group	Böhler angle (^o^)	Gissane angle (^o^)	Calcaneal length (mm)	Calcaneal width (mm)	Calcaneal height (mm)
Preoperative	19.1 ± 6.1	104.1 ± 16.6	60.1 ± 5.0	37.0 ± 3.0	31.6 ± 2.5
Three months postoperative	30.6 ± 6.7*	119.8 ± 6.4*	64.8 ± 8.0	33.1 ± 3.0*	36.9 ± 1.7*
Last follow-up	30.6 ± 6.6*	119.7 ± 6.4*	66.6 ± 10.3*	33.0 ± 3.0*	37.1 ± 1.8*

There was no statistical difference between the 3-month postoperative group and the last follow-up group

*p < 0.01, compared with the preoperative group

**Table 3:** Function outcome scores

Maryland score	Mean ± SD	Excellent (%) (90–100)	Good (%) (75–89)	Fair (%) (50–74)	Poor (<50)
Total	88.1 ± 8.8	11 (61.1)	4 (22.2)	3 (16.7)	0 (0)
Sanders II	88.1 ± 8.8	8 (57.1)	4 (28.6)	2 (14.3)	0 (0)
Sanders III	87.8 ± 10.1	3 (75.0)	0 (0)	1 (25.0)	0 (0)

## Discussion

The use of open reduction through a minimally invasive sinus tarsi approach and stabilization with a locking plate is now a widely accepted treatment for calcaneal fractures [5–7, 15, 16]. However, all the currently used locking plates are in one piece, which requires a large working space for placement, leading to the necessity of sufficient exposure of the lateral wall of the calcaneus and even establishment of assisted incision [7]. This may increase the injuries to the soft tissues and the risk of wound complications. In addition, simultaneous fixation of multiple fracture blocks also induces difficulty in operation, easily causing poor anatomic reduction [5, 17]. To minimize the above problems, we newly designed and patented a combined plate in 2014. Our plate can be inserted through the sinus tarsi incision by using only appropriate dissection of subcutaneous tissues and the periosteum, without the need for the creation of a complete subcutaneous tunnel [7]. The streamlined shape of the head end can reduce the injuries to the surrounding soft tissues, whereas the chimeric structure of the tail end with a coupling screw can form a triangle support with the anterior and posterior calcaneus, which are respectively fixed with three locking screws, thus providing a rigid fixation and avoiding reduction loss. Furthermore, the angle between the forearm and the rear arm was set to 130°, with reference to the normal ranges of the Böhler angle (20–40°) and Gissane angle (110–140°), as well as the biomechanical characteristics of the calcaneus, by which the maximum area can be fixed under sufficient axial stress resistance and the risk of plate breakage can be decreased. We hypothesized that our combined plate may be effective and safe for the management of calcaneal fractures.

The present study aimed to preliminarily confirm the treatment outcomes of the plate for calcaneal fractures, by retrospectively collecting our clinical data, which, to our knowledge, have not been reported. As expected, our results demonstrated that the Böhler angle, Gissane angle, calcaneal height, width, and length were all significantly corrected postoperatively, without any accompanying postoperative wound complications and implant loosening during the follow-up. Our results seemed to be superior to those of the study by Kikuchi et al. [10], in which the sinus tarsi approach was also used: three of their cases (13.6%) had superficial wound infections, and no statistical differences were present in the Gissane angle, calcaneal height, and calcaneal length between preoperatively and postoperatively. Moreover, in the study of Basile et al., three patients (7.9%) who underwent the sinus tarsi approach failed to achieve anatomic reduction [5]. These findings indicated the effectiveness and safety of our plate for calcaneal fractures.

Better reduction of the calcaneus may result in better recovery of foot function. As anticipated, our findings indicated that the mean MFS reached 88.1 ± 8.8, with an excellent and good rate of 83.3% (15 of 18). This result seemed to be comparable with, but not higher than, that of previous studies [6, 7]. We believe that this may be attributed to the small sample size (18 vs 33) and shorter follow-up (9.3 ± 3.7 vs 21 ± 8.9 months) [2].

In line with the simple and convenient procedure for placing our plate, the operative time was obviously reduced in our study when compared with previous studies (64.4 ± 8.0 vs 69 ± 14.6 min [2]; 64.4 ± 8.0 vs 122.15 ± 8.32 [5]), which may possibly further decrease the infection rate and promote fracture healing, resulting in a shorter hospital stay. Furthermore, it had also been demonstrated that the radiation exposure time in our study was dramatically shorter than that in the study by Cao et al. [2] (13.0 ± 3.5 s vs 3.6 ± 0.5 min). Thus, the use of our plate may prevent radiation-associated toxicity in surgeons and patients, further indirectly demonstrating the safety of our procedure.

The severity of closed calcaneal fractures is known to be negatively correlated to the subsequent foot function reduction and the quality of life after open reduction and internal plate fixation [18]. Thus, we also compared the MFS between Sanders types II and III calcaneal fractures. However, the results indicated a similar outcome, demonstrating that our self-designed plate may be equally suitable for these two types of fractures. However, further studies are still needed to prove our conclusion because of the small number of included patients with Sanders type III calcaneal fractures.

By summarizing our clinical experience, we consider that the indications for our plate treatment are as follows: (1) Sanders type II or simple type III fracture; (2) uneven articular surface, with displacement ≥1 mm; (3) loss of calcaneal height >1.5 cm and increase of calcaneal width >1 cm; (4) Böhler angle ≤20°, Gissane angle ≤100° or ≥130°; or (5) calcaneal varus angle ≥50° and eversion ≥10°. However, there are still some limitations in our technique. For example, percutaneous cannulated screw fixation is still needed as an assisted reduction for the treatment of Sanders type III calcaneal fractures with involvement of the sustentaculum tali or extra-articular calcaneal fractures (e.g. beak/avulsion fracture of the calcaneal tuberosity). Further optimization of our combined plate is still necessary.

Our study has some limitations. First, this was a retrospective review and the patients were not randomized to receive our surgery. Thus, the choice of surgery might be biased by surgeon preference and patient factors. Second, we did not have a control group, which made our comparison inconclusive. Third, our sample size of 18 was relatively small and the follow-up was short, which may have led to the underestimation of the complication rate and long-term reduction effect. Fourth, we preliminarily assessed the biomechanical properties of our combined plate but did not conduct a confirmation with a cadaver study or with bone models. Therefore, further investigation is still essential to obtain a more precise efficacy evaluation, by conducting a biomechanical study in human cadavers or bone models, or by means of a clinical study with a large sample size, longer follow-up time, and a randomized control (with other plates).

## Conclusions

Our present study suggests that the minimally invasive sinus tarsi approach with our combined plate may be a safe and effective alternative for the operative treatment of type II and simple type III calcaneal fractures, with rare postoperative complications and excellent reduction capacity.

## Additional files

Additional file 1: Additional movie file. (MEP 2 kb)
Additional file 2: Table S1. Case serials. (DOCX 71 kb)

## Abbreviations

CT: Computed tomography
MFS: Maryland foot score
