# Supplementary material for: Minimally invasive (sinus tarsi) approach for calcaneal fractures
Source: J Orthop Surg Res. 2016 Dec 23;11:164. doi: 10.1186/s13018-016-0497-4 (PMC5180402; doi:10.1186/s13018-016-0497-4)
Supplement: Additional file 2: Table S1. — Case serials. (DOCX 71 kb) [file 13018_2016_497_MOESM2_ESM.docx]

**Table S1 Case serials**

| Case | Sex | Age  (y) | Side | Injury mechanism | Complicated other fractures | History | Sanders | Interval from injury to surgery (d) | Operative time  (min) | Radiation (s) | Hospital stay (d) | Follow up (m) | Healing  time  (d) |
| --- | --- | --- | --- | --- | --- | --- | --- | --- | --- | --- | --- | --- | --- |
| 1 | M | 52 | L | Falling from a height |  | Smoking | IIC | 4 | 60 | 10 | 7 | 6 | 103 |
| 2 | M | 45 | L | Falling from a height |  |  | IIA | 7 | 70 | 12 | 9 | 13.5 | 90 |
| 3 | M | 66 | R | Falling from a height |  | Smoking, diabetes | IIA | 3 | 80 | 15 | 10 | 6 | 88 |
| 4 | M | 52 | R | Falling from a height |  |  | IIIAB | 3 | 60 | 12 | 5 | 11 | 97 |
| 5 | M | 50 | L | Falling from a height | Lumbar vertebral |  | IIA | 2 | 70 | 13 | 6 | 17 | 82 |
| 6 | M | 32 | R | Falling from a height | Ankle |  | IIB | 3 | 50 | 10 | 10 | 6 | 78 |
| 7 | M | 47 | L | Falling from a height |  |  | IIIAB | 8 | 60 | 8 | 10 | 14 | 77 |
| 8 | M | 50 | R | Falling from a height |  | Smoking | IIB | 8 | 60 | 11 | 11 | 14 | 83 |
| 9 | F | 52 | L | Traffic accidents |  | Diabetes | IIB | 4 | 65 | 17 | 7 | 12 | 95 |
| 10 | M | 46 | R | Falling from a height |  |  | IIA | 3 | 60 | 13 | 5 | 9 | 93 |
| 11 | M | 50 | R | Falling from a height |  |  | IIB | 3 | 65 | 16 | 8 | 6 | 76 |
| 12 | M | 41 | R | Falling from a height |  |  | IIA | 8 | 75 | 18 | 13 | 6 | 84 |
| 13 | M | 32 | R | Falling from a height |  |  | IIIAC | 9 | 75 | 21 | 10 | 13 | 75 |
| 14 | F | 61 | R | Others |  | Smoking | IIA | 2 | 60 | 16 | 8 | 6 | 79 |
| 15 | F | 62 | L | Falling from a height |  | Smoking | IIA | 7 | 70 | 9 | 10 | 8 | 98 |
| 16 | M | 62 | R | Stroke | Clavicular |  | IIC | 4 | 55 | 9 | 11 | 8 | 105 |
| 17 | M | 43 | L | Traffic accidents |  | Smoking, alcohol consumption | IIBC | 3 | 55 | 13 | 9 | 6 | 92 |
| 18 | F | 65 | R | Falling from a height |  |  | IIA | 4 | 70 | 11 | 11 | 6 | 113 |

| Case | Preoperatively | | | | | Three months after operation | | | | | Last follow up | | | | | Maryland  score |
| --- | --- | --- | --- | --- | --- | --- | --- | --- | --- | --- | --- | --- | --- | --- | --- | --- |
|  | Bohler  (o) | Gissanne  (o) | Length (mm) | Width (mm) | Height (mm) | Bohler (o) | Gissanne  (o) | Length (mm) | Width (mm) | Height (mm) | Bohler  (o) | Gissanne  (o) | Length (mm) | Width (mm) | Height (mm) |  |
| 1 | 16.75 | 144.97 | 58.23 | 38.59 | 33.38 | 33.04 | 120.49 | 70.28 | 35.54 | 35.6 | 33.29 | 120.67 | 70.28 | 35.54 | 35.6 | 95 |
| 2 | 31.9 | 132.83 | 62.33 | 39.35 | 34.52 | 35.91 | 118.58 | 67.53 | 39.28 | 38.57 | 34.92 | 117.97 | 67.83 | 39.3 | 38.6 | 88 |
| 3 | 18.07 | 132.58 | 57.44 | 36 | 28.67 | 36.31 | 124.59 | 65.43 | 33.33 | 35.68 | 36.31 | 124.59 | 65.43 | 33.33 | 35.77 | 96 |
| 4 | 32.33 | 109.56 | 65.76 | 38.97 | 30.02 | 37.8 | 122.93 | 69.44 | 35.65 | 35.48 | 37.9 | 123 | 69.5 | 36.9 | 35.5 | 92 |
| 5 | 23.63 | 93.27 | 63.2 | 34.33 | 33.31 | 33.94 | 128.22 | 68.89 | 29.63 | 34.12 | 33.45 | 127.1 | 68.9 | 29.8 | 34.2 | 95 |
| 6 | 19.04 | 86.08 | 59.11 | 40.64 | 31.46 | 42.37 | 111 | 63.2 | 29.63 | 39.1 | 42.37 | 110.43 | 62.2 | 29.63 | 39.23 | 91 |
| 7 | 13.07 | 102.14 | 61.15 | 42.61 | 32.83 | 41.03 | 126.21 | 69.73 | 32.99 | 35.89 | 41 | 126.18 | 69.8 | 33 | 36.8 | 90 |
| 8 | 14.7 | 97.62 | 64.77 | 38.81 | 29.83 | 26.64 | 109.07 | 71.2 | 36 | 37.92 | 27.7 | 109 | 71.13 | 36.44 | 37.95 | 93 |
| 9 | 20.62 | 112.28 | 63.25 | 33.91 | 34.28 | 29.28 | 121.51 | 66.7 | 32.66 | 36.76 | 29.3 | 121.31 | 66.65 | 31.45 | 35.8 | 98 |
| 10 | 23.04 | 99.78 | 55.53 | 37.54 | 32.78 | 33.39 | 121.43 | 61.87 | 35.16 | 37.92 | 33.4 | 122.34 | 92.99 | 35.18 | 38.02 | 85 |
| 11 | 22.38 | 94.66 | 64.83 | 32.17 | 34.2 | 34.19 | 117.15 | 69.17 | 26.66 | 37.15 | 32.59 | 117.15 | 69.17 | 28.22 | 39.02 | 87 |
| 12 | 17.37 | 97 | 52.33 | 37.77 | 32.01 | 23.17 | 124.4 | 60.77 | 35.86 | 37.19 | 23.17 | 124.4 | 61.96 | 35.86 | 37.19 | 69 |
| 13 | 18.54 | 91.39 | 50.97 | 34.32 | 27.66 | 22.9 | 115.23 | 63.85 | 31.22 | 34.93 | 23.8 | 115.2 | 63.85 | 31.28 | 34.93 | 73 |
| 14 | 10.88 | 103.83 | 55.3 | 34.31 | 30.79 | 20.24 | 123.18 | 64.33 | 30.82 | 38.52 | 20.22 | 123.18 | 64.33 | 29.93 | 38.52 | 78 |
| 15 | 19.16 | 100.67 | 62.67 | 32.19 | 35.86 | 28.86 | 128.13 | 70.23 | 31.53 | 38.21 | 28.9 | 128.51 | 71.18 | 31.6 | 38.56 | 94 |
| 16 | 13.22 | 94.46 | 54.28 | 36.12 | 29.15 | 22.35 | 105.25 | 62.76 | 31.47 | 39.62 | 21.9 | 105.11 | 62.7 | 30.82 | 39.62 | 74 |
| 17 | 10.28 | 90.63 | 60.97 | 40.2 | 28.44 | 24.67 | 117.88 | 65.73 | 33.2 | 37.22 | 24.67 | 117.88 | 65.73 | 32.2 | 38.02 | 96 |
| 18 | 18.12 | 90.02 | 69.58 | 38.54 | 28.78 | 25.06 | 120.37 | 35.6 | 34.41 | 34.06 | 25.16 | 121.02 | 35.64 | 34.41 | 33.77 | 91 |
